# A multi-objective approach for timber harvest scheduling to include management of at-risk species and spatial configuration objectives

**DOI:** 10.1371/journal.pone.0302640

**Published:** 2024-10-25

**Authors:** Max D. Jones, Angela Larsen-Gray, Stephen P. Prisley, Holly L. Munro, Elizabeth A. Hunter

**Affiliations:** 1 Department of Fish and Wildlife Conservation, Virginia Tech, Blacksburg, VA, United States of America; 2 National Council for Air and Stream Improvement, Inc., Blacksburg, VA, United States of America; 3 National Council for Air and Stream Improvement, Inc., Athens, GA, United States of America; 4 U.S. Geological Survey, Virginia Cooperative Fish and Wildlife Research Unit, Department of Fish and Wildlife Conservation, Virginia Tech, Blacksburg, VA, United States of America; Western Carolina University, UNITED STATES OF AMERICA

## Abstract

Sustainable forestry typically involves integration of several economic and ecological objectives which, at times, may not be compatible with one another. Multi-objective prioritization via harvest scheduling programs can be used to elucidate these relationships and explore solutions. One such program is a spatially explicit harvest scheduler that adopts the Metropolis-Hastings algorithm to iteratively find management solutions to achieve multiple objectives (Habplan). Although this program has been used to address forest management scheduling and simulation-based tasks, its utility is constrained by time-intensive data preparation and challenges with incorporating spatial configuration objectives. To address these shortcomings, we introduce an open-source software package, HabplanR, streamlines data preparation, sets parameters, visualizes results, and assesses spatial components of ecological objectives. We developed four example objectives to incorporate into a multi-objective management problem: habitat quality indices for three species “types” (open, closed, and intermediate-canopy-associated species), and harvested pine pulpwood (revenue). We demonstrate the utility of this package to find management schedules that can accommodate potentially conflicting habitat needs of species, while achieving economic targets. We produced 100 software runs and prioritized individual objectives to select four management schedules for further comparisons. We compared outcome differences of the four schedules, including a spatial comparison of two high performing schedules. The software package makes costs and benefits of different schedules explicit and allows for consideration of the spatial configuration of management outcomes in decision-making.

## Introduction

Forests can be managed for recreation, wildlife conservation, carbon sequestration, economic objectives (among others), or a combination of multiple objectives; driven largely by landowner objectives [1]. In fact, forest certification standards, such as American Tree Farm System (ATFS; [2]), Sustainable Forestry Initiative (SFI; [3]), and Forest Stewardship Council (FSC; [4]), also highlight the need for forest landowners and managers to consider multiple objectives. Forest certification programs adhere to a set of principles that reflect a commitment to provide certain benefits to society, including conservation of biological diversity [5]. As an example, the recently updated 2022 SFI Forest Management Standard [6] includes requirements to protect water quality, biodiversity, wildlife habitat, species at risk, forests with exceptional conservation value, carbon storage, and climate smart forestry to promote sustainable forestry at stand and landscape scales.

Some forest management objectives may be incompatible at times, particularly when attempting to prioritize economic and conservation goals. However, there may be situations in which minor opportunity costs to one objective (e.g., timber production) could have a substantial benefit to another objective (e.g., conservation of at-risk species [7]). Multi-objective prioritization approaches can be used to inform best strategies for complex management needs, particularly for management options that require conservation of biological diversity, valuable economic services, or that address emerging issues such as climate change [8–10]. There are several methods and accompanying programs that can be used to perform multi-objective or multi-species management. For example, Martin et al. [11] used a linear programming method to demonstrate that protected area networks could be expanded to areas important for boreal caribou (*Rangifer tarandrus*), other at-risk species, and climate objectives. McGowan et al. [12] used a stochastic dynamic programming method to incorporate management actions, population models, and objective weighting to optimize a multi-species adaptive management plan. However, in the case of conflicting objectives (i.e., one objective benefits only at the cost to another) or many objectives, mathematical optimization through dynamic programming can either be infeasible or impractical for forest managers due to time and computing constraints [13].

Multi-objective approaches more effectively address species conservation objectives when they incorporate spatial arrangement of landscapes [14–16]. In multi-objective forest planning, the spatiotemporal attributes of forest harvesting schedules can directly affect landscape structural characteristics, such as edge/interior forest area, patch size and number, forest stand age and size, and location of infrastructure development projects (e.g., road construction) [14–17]. Increasing connectivity of disjunct landscape patches may help sustain and bolster certain area-sensitive at-risk populations [18, 19]; whereas other species may benefit from the finer-scale heterogeneity that harvesting creates [20, 21]. Creating management plans and landscape arrangements that address multiple objectives is particularly challenging when objectives (e.g., forest area, yield) conflict in their optimal solutions [9, 11]. In these cases, conflicting objectives cannot be maximized simultaneously. Therefore, the ability of forest managers to investigate possible costs and benefits of various strategies may increase effectiveness of management plans [22]. Using criteria and indicators of management success or relative weighting of objectives, often led by input from multiple partners, may support decision-making [23].

Ignoring conflicting objectives and prioritizing single-objective management strategies can sometimes cause unforeseen consequences in other objectives [24–26]. For example, previous research in boreal forests has shown that it was not possible to maximize carbon storage and biodiversity objectives when prioritizing revenue from timber harvest [24]. Harvest scheduling programs (e.g., LANDIS [27]) have historically focused on forest economics but can be used to address additional objectives. One such program is Habplan, which is a spatially explicit harvest scheduling program, written in Java, that utilizes the Metropolis-Hastings algorithm to assess the effect of iteratively substituted forest management regimes on specified objectives to discover optimal (or near-optimal) solutions that can maximize each objective [28]. This program produces a schedule that reflects a set of user-defined management regimes assigned to each forest stand (polygon) and will produce a nearly infinite number of possible schedules, unless it reaches a maximum iteration input or is stopped by a user once goal functions are attained [29–31]. The program has been applied to several forest management scenarios that have biodiversity and conservation-related objectives [28, 31–33]. Simulation-based tools that iteratively search for possible solutions offer the advantage of allowing land managers to evaluate merits of different management regimes in maximizing different objectives; including sustainable forestry practices and forest certification.

Despite the utility of Habplan as an efficient harvest scheduling program, use is currently limited due to time-intensive data preparation and challenges with incorporating spatial configuration objectives. We developed the custom R package *HabplanR* to streamline production of the program files and setting objective parameters within R. The custom package applies spatial patch analysis across a landscape to provide managers with a decision-support tool for complex spatial forestry issues. We addressed two main objectives: 1) Demonstrate how a harvest scheduling program’s algorithm can be used to create management schedules to manage multiple species with complex and conflicting habitat needs, in addition to economic yield; and 2) Highlight utility of the an open-source software package to complement the harvest scheduling program. To address these objectives, we provide a workflow for using a multi-objective approach via Habplan software and further adopt readily available and reproducible spatial analyses to aid decision-making for managers facing complex forest management decisions–which we embedded into *HabplanR* functions. We provide a step-by-step guide for tackling forest management decisions when faced with conflicting management objectives. We introduce a real-world problem and accompanying dataset from several managed pine (*Pinus* spp.) landscapes in the southeastern United States (U.S.).

## Materials and methods

### Overview

**Fig 1:**
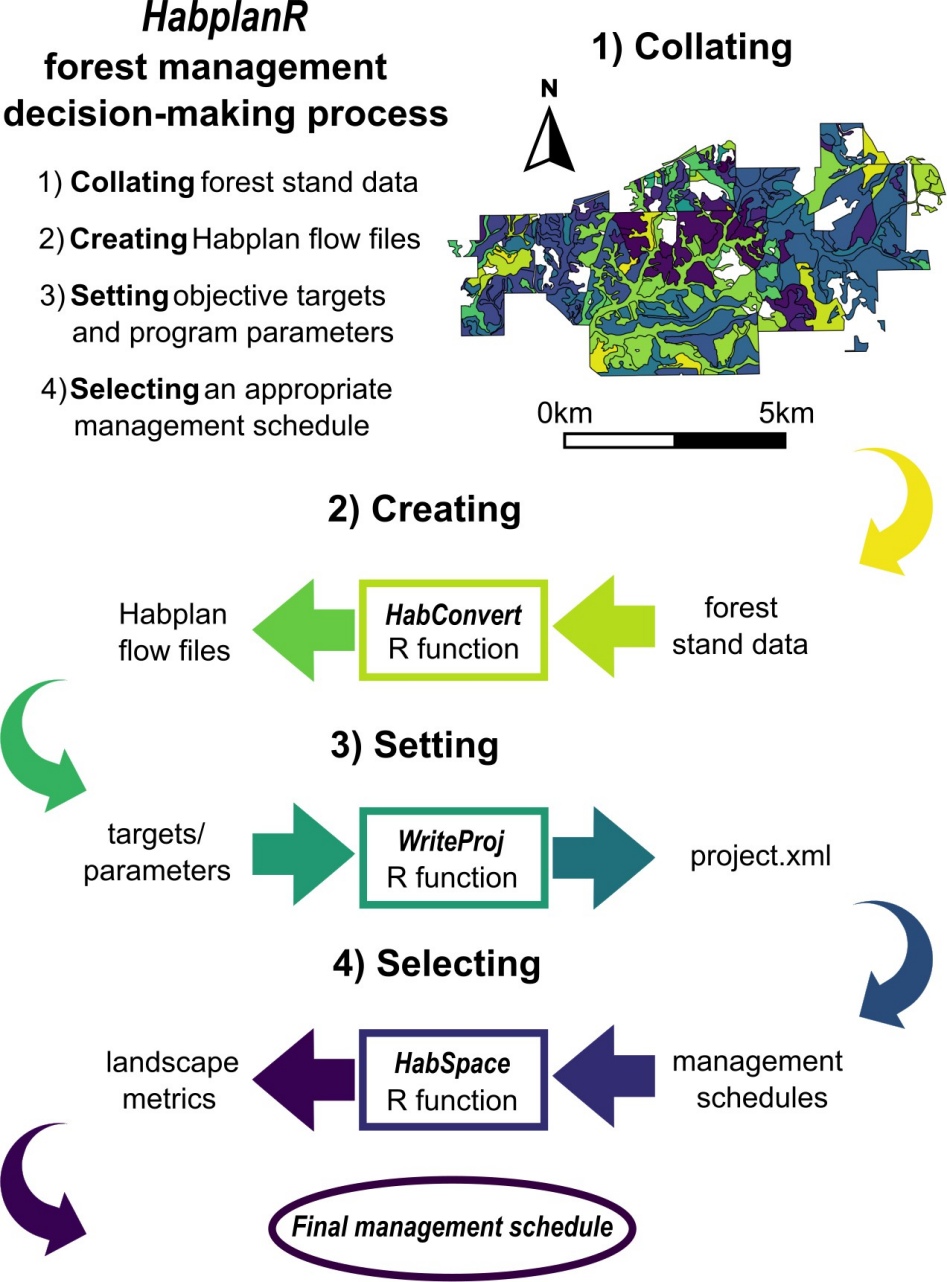
Flow diagram of forest management decision-making process using Habplan and *HabplanR*. Bold text numbered 1–4 represent the four main steps of the forest management decision-making process. Colored rectangles depict when a specific *HabplanR* function is run during the process, and text before inward-facing arrows show R function inputs, and text after outward-facing arrows represent R function outputs. Colors in the provided map highlight stand boundaries.

We considered four objectives to meet throughout 35, three-year periods (105 years total) across 505 forest stands that composed a theoretical managed forest landscape, including maintaining and creating conditions for three species *types* (open, closed, and intermediate-canopy-associated wildlife species), and one measure of economic yield (harvested pine pulpwood). We used three-year periods to reduce data demands and thus computational power, and annual predictions, or other used-defined periods can be used. Our decision-making process had four main steps: 1) collating forest stand data; 2) creating Habplan flow files; 3) setting objective targets and program parameters; and 4) selecting an appropriate management schedule (Fig 1).

### Case study–Pine forests in the southeastern U.S.

#### Step 1: Collating forest stand data

Southern U.S. longleaf pine (*Pinus palustris*) forests were historically characterized by relatively open canopies with varying tree age classes, supporting a high diversity of plant and wildlife species [34–36]. European settlement was followed by persistent fire suppression throughout the region, which resulted in these biodiverse open canopy pine communities transitioning to hardwood-encroached, closed-canopy forests that could not support many disturbance-adapted wildlife species [37]. Today, pine forests are actively managed, predominantly for timber, but increasingly plans incorporate specific wildlife conservation goals, often using a multitude of management strategies such as prescribed burning, thinning, and herbicide treatment, alone or in combination [38, 39]. Forest management is particularly important for some open canopy pine associated at-risk species, especially disturbance-adapted species that are often state or federally listed as threatened or endangered and have experienced considerable declines within North America [40, 41].

Nearly 90% of southeastern U.S. pine forests are privately owned and primarily managed for timber production, which includes harvesting both pulpwood, commonly used in paper and fiberboard, and sawtimber, often used for higher-value products like lumber for construction [42]. The economic importance of working pine forests has helped create a sustainable forest industry in the southeastern United States. Forest sustainability, due to societal expectations, is now a driver for forest certification initiatives and thus, it has become more common for private forest owners to consider multiple objectives, including wildlife conservation, alongside yield targets [7], thereby creating a need for tools for multi-objective decision-making.

**Fig 2:**
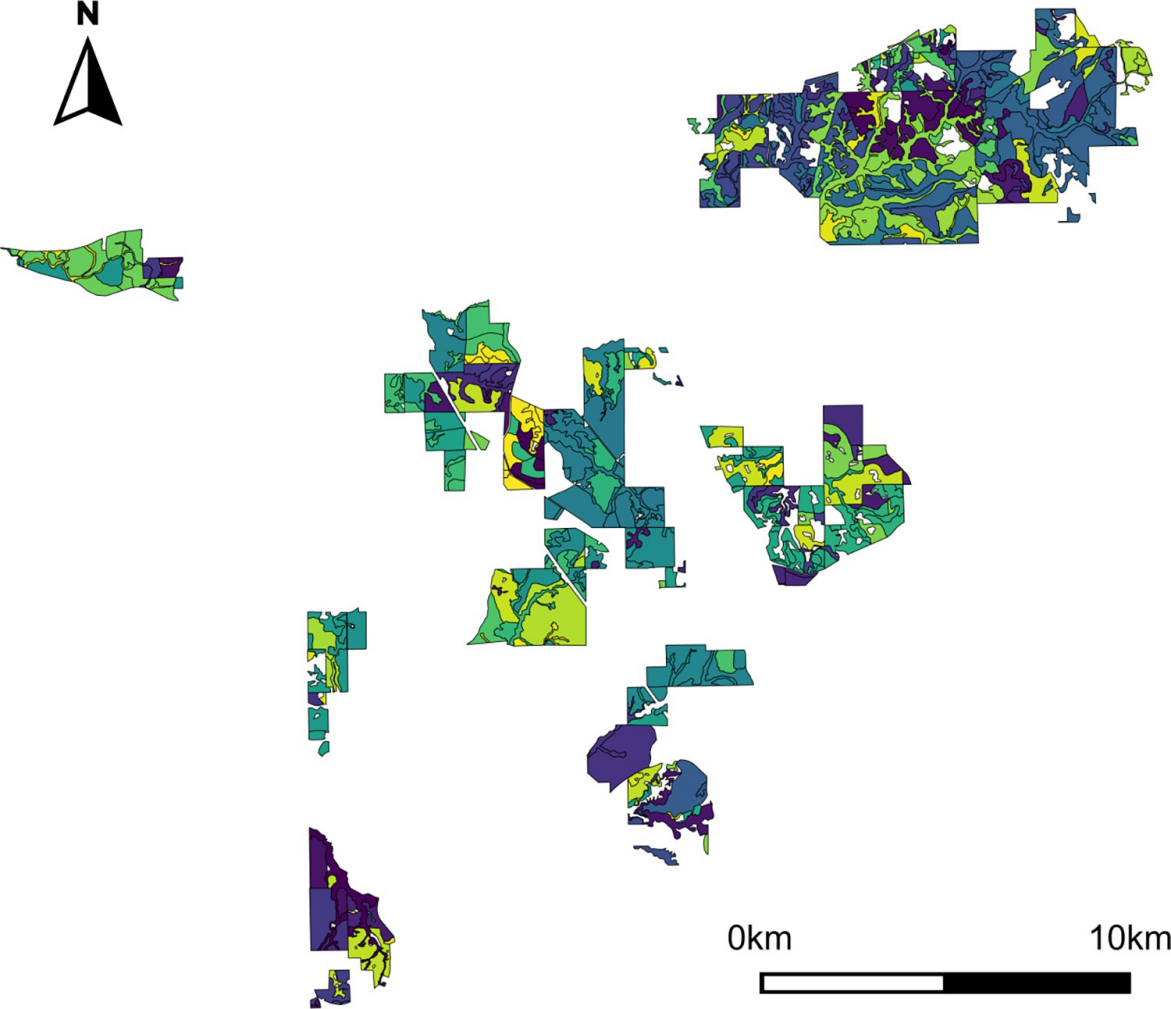
Study site map highlighting the 505 forest stands that were incorporated into the Habplan harvest scheduler. Colors in the provided map highlight stand boundaries. Map lacks a coordinate reference system for anonymity.

To demonstrate use of the custom package for multiple objective management, we obtained stand inventory data representing 505 forest stands in a privately-owned forest in the southeastern United States (specific location withheld for anonymity; Fig 2), covering 17,774 acres with a mean stand area of 35.2 acres (0.48–763.2 acres). We received stand data in acres, and therefore maintained the use of acres throughout to reflect our case study. We projected each stand into the future with a planning horizon of 35, three-year periods (105 years), using the Forest Vegetation Simulator (FVS) growth model [43, 44]. We projected stands under 10 unique management regimes (S1 Table), which produced a list of all possible stand/regime combinations and their corresponding outputs. We selected these regimes due to the combination of forest stands available throughout the study site, and did not focus primarily on timber-production related objectives. Instead, we included management options that either replaced existing loblolly pine (*Pinus taeda*) stands with longleaf pine stands over time, and managed these pine stands to achieve large-diameter conditions. Regimes and corresponding outcomes we used may differ for forests that are focused on timber production. Our FVS growth model simulated basal area (m^2^/ha), tree species and size class per area unit, and volume (m^3^) of wood harvested each period (3 years). From these data, we derived a single wood flow to represent forest harvesting and revenue: tons of pine pulpwood per acre. We used this measure purely for example; any other forest productivity metric could be used, including in combination (e.g., harvested pine pulpwood and sawtimber production).

**Fig 3:**
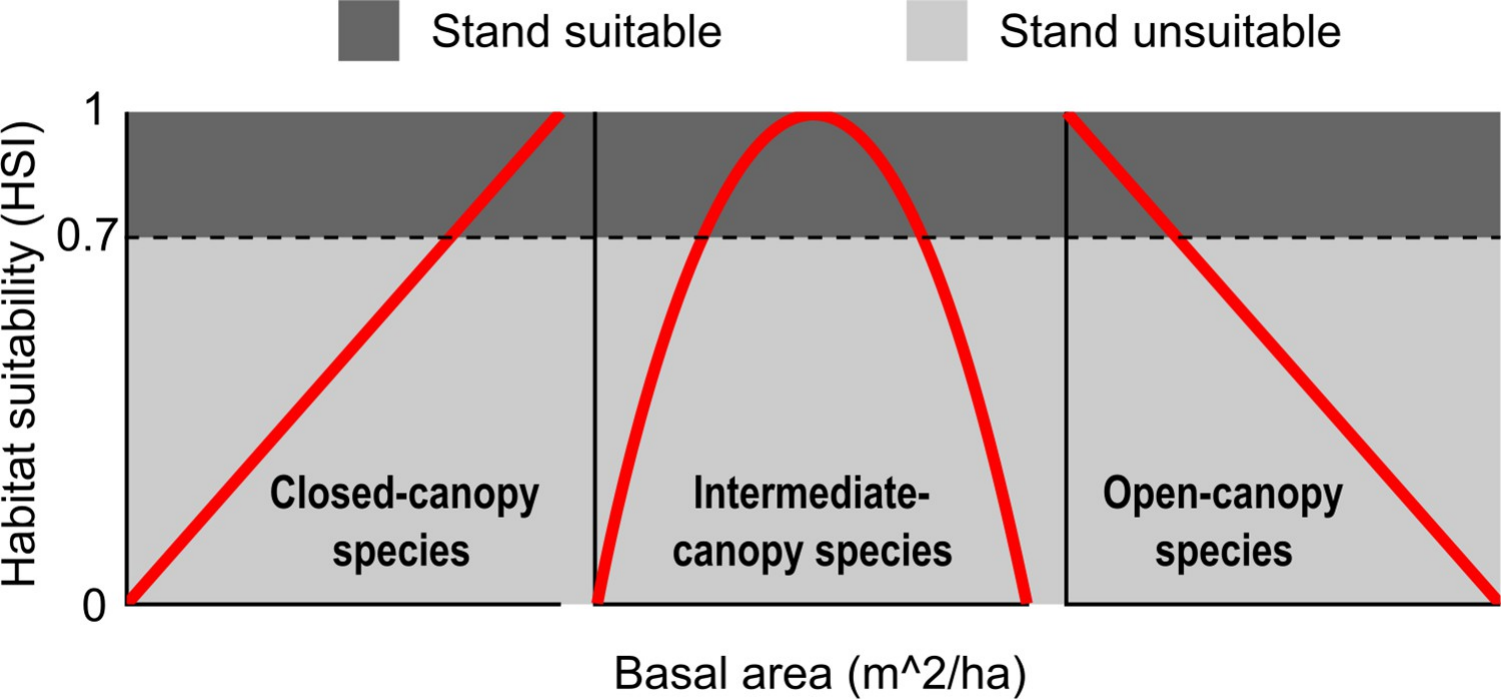
Representations of three species “types” with divergent habitat requirements. Red line demonstrates relationship between basal area and habitat. Black dashed line depicts Habitat Suitability Index (HSI) threshold of 0.7 used to determine if a stand hosted habitat or non-habitat (can be altered for each species, see Step 2). Left: Closed-canopy-associated species; Middle: intermediate-canopy-associated species; Right: Open-canopy-associated species.

To demonstrate how a harvest scheduling program can be used to identify management strategies for conflicting objectives, we developed three objective functions for species “types” with different habitat needs. We developed simple Habitat Suitability Indices (HSI), which are habitat quality indices, but we use HSI to keep consistent language with previous literature. Our HSIs differed in their relationship with basal area, which we used as a proxy for canopy openness (Fig 3). Thus, our HSI would not distinguish between highly different cover types with similar basal area values and was used only as an example, and our given species below act as representations of species to which these values may apply. More complex habitat relationships could be developed (any combination of forest traits that are present in the forest projection dataset), but we use a single indicator to simplify presentation of potential outcome differences caused by different regimes. The first HSI was a positive linear relationship with basal area, representing a species associated with young, closed-canopy pine forest such as Swainson’s warbler (*Limnothylpis swainsonii*), a species of conservation concern that has been found using loblolly pine stands as breeding habitat [45, 46]. The second HSI included a quadratic relationship with basal area, representing a species requiring an intermediate basal area for habitat, such as southeastern pocket gophers (*Geomys pinetis* [47]). The final HSI included a negative linear relationship between HSI and basal area, representing an open-canopy-associated species, such as gopher tortoises (*Gopherus polyphemus*), or northern bobwhite (*Colinus virgnianus)*.

We produced HSI values using the *HSIcalc* function included in *HabplanR*. We defined each of the above relationships as an R function (see the *HabplanR Vignette* for examples; public version available at: [48]) and input these functions into *HSIcalc* using the *equation* argument. We provided the FVS growth data using the *std*.*data* argument. The function applies user-defined equations and data from the growth model to calculate an HSI value for each combination of stand ID, management regime, and time period, and then creates a new column in the input *std*.*data* data frame. Users have the option to select true or false on the *logistic* argument of the *HSIcalc* function (default = true, *logistic = T*). The *logistic* argument lets the function know whether to apply a logistic function to the calculated value thus bounding values between 0–1, which is typically appropriate for HSI values.

#### Step 2: Creating Habplan flow files (.dat)

Previously, data preparation for input into Habplan took considerable time. Prior to engaging with Habplan, forest stand conditions needed to be projected into the desired future timeframe (e.g., using a growth model as above). Once these data were projected, the resulting data frames needed to be converted into Habplan-specific files, which was completed manually. These input and output files are referred to as *flow* files (also referred to as flows, or flow components) and contain each stand ID, regime acting on the stand, projected time period (e.g., year), and projected output/yield.

*HabplanR* contains the function *HabConvert*, which can be used to convert data frames containing stand growth data (e.g., FVS growth model data) into Habplan flow files much faster than before. *HabConvert* requires input of two.csv format files: 1) *std*.*data* contains all the projected growth data for each stand with each column indicating a specific objective (e.g., pulp pinewood yield, dollar revenue), and 2) *std*.*info*, a list of matching stand IDs with the corresponding stand area. Additionally, the function requires the number of projected time periods using the *nyear* argument. Our case study projected stands for 35 three-year periods (*nyear = 35*).

The *HabConvert* function provides a final optional argument for flow file creation, *HSI*. The *HSI* argument is input as a numerical threshold between 0 and 1, which indicates if stand conditions are suitable or unsuitable (i.e., produced habitat and thus the stand acreage was included or not included in projected habitat area; Fig 3). We assigned the *HSI* argument as 0.7 (*HSI = 0*.*7*), so only an HSI projection greater than 0.7 (for the projected stand and time period combination) is classified as habitat. However, this can be set to a different level, allowing for user flexibility.

#### Step 3: Setting objective targets and program parameters

Targets for the various objectives in a Habplan modeling exercise are defined as levels of output/flow across time periods. Objective targets and parameters are assigned via a *Flow Form* in the Habplan program. A list of flow form parameters that can be edited, including their function, can be found in the Habplan User Manual [49]. We provide a means to edit these as R objects within the *HabplanR Vignette* [48]. For the purpose of our case study, we highlight only a few of these options that we edited to produce multiple outputs from Habplan for comparison.

1. *Model*: A combination of time periods and objective targets. Years and corresponding targets are separated by commas and these combinations are separated by semicolons. For example, `1, 1000; 2, 2000`shows that the target for time period one is 1,000 (of that objective unit; e.g., tons harvested), and the target is increased to 2,000 in time period two.
2. *Thlo*: The allowable negative deviation from the objective target (e.g., area of habitat, tons of harvested pine pulpwood).
3. *Thhi*: The allowable positive deviation from the objective target.

**Fig 4:**
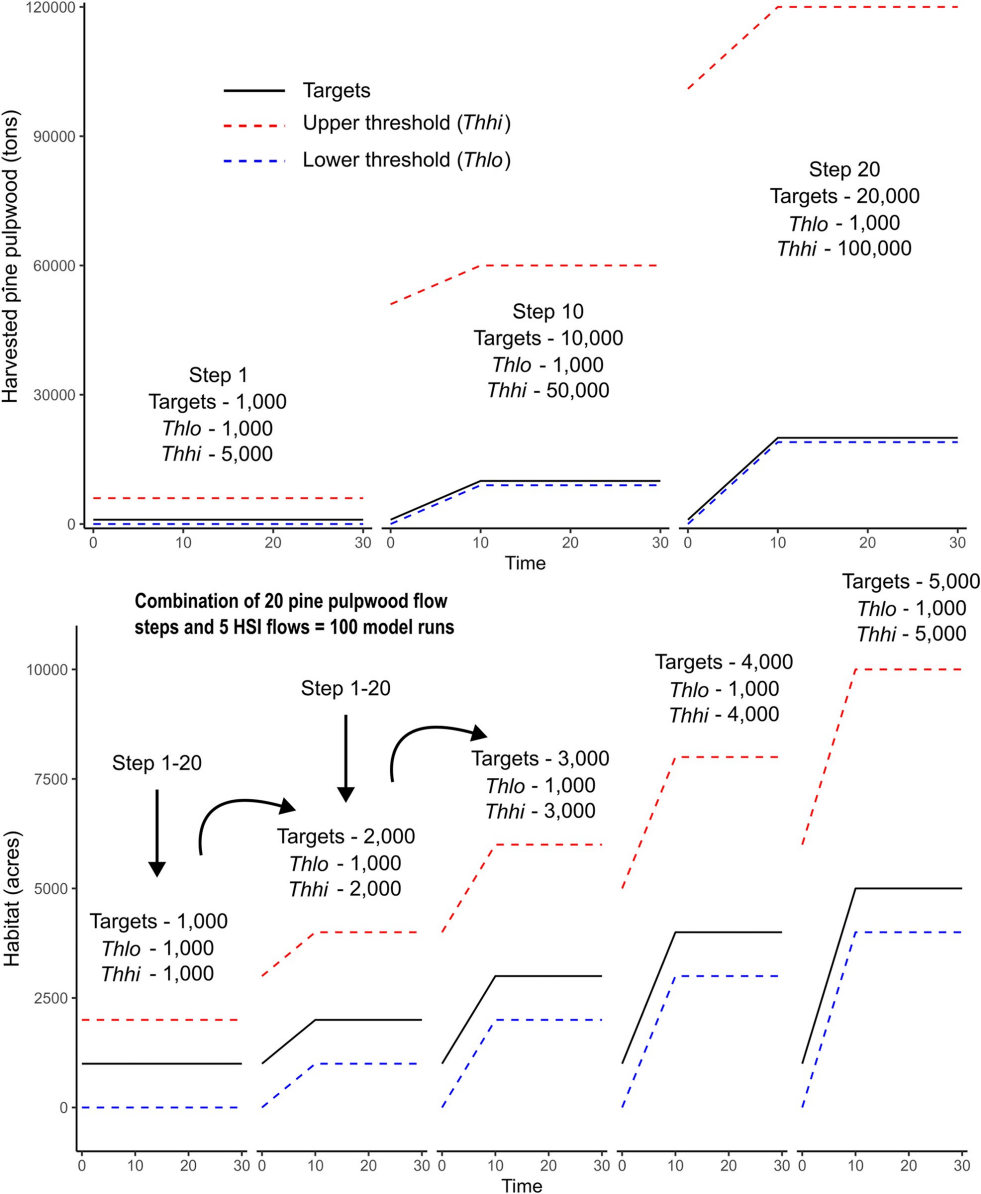
A schematic of the relationships between model targets and upper (*Thhi*) and lower (*Thlo*) thresholds (i.e., how much the target is able to positively and negatively deviate respectively). The upper panel represents parameters set for pine pulpwood flows, where 20 “steps” were parameterized (only three steps shown for simplicity). The lower panel shows the five sets of parameters set for the HSI flow, which are run in combination with the 20 pine pulpwood steps for 100 model runs (S2 Table).

Using the above parameters, we specified objective targets (*model*) for years 10, 20, and 30 for each flow component, along with an upper (*thhi*) and lower (*thlo*) threshold for which those targets could positively and negatively deviate respectively (Fig 4). To demonstrate how Habplan can be used to provide alternative management strategies when different objectives have varying target levels, we created 100 model runs with variations in *model*, *thhi*, and *thlo* for each flow (S2 Table). We did this as an example of how to produce and examine varying solutions, but values of *model*, *thhi*, and *thlo* could be changed to fit alternative situations. For simplicity, we set the same parameters for the three HSI flows that started at a value of 1,000 acres for targets, *thhi* and *thlo* (*model = “10*,*1000; 20*,*1000; 30*,*1000”*, *thlo = 1000*, *thhi = 1000)*. We maintained the *model* targets for 20 steps of Habplan, in which we only changed the targets, *thhi* and *thlo*, for the pine pulpwood flow component. We then increased each pine pulpwood flow target for years 10, 20, and 30 by 1,000 tons, and *thhi* by 5,000 tons for each step (e.g., the first 3-year period *model = “10*,*1000; 20*,*1000; 30*,*1000”*, *thlo = 1000*, *thhi = 5000*; the second 3-year period: *model = “10*,*2000; 20*,*2000; 30*,*2000”*, *thlo = 1000*, *thhi = 10000*). After 20 steps (Fig 4), we reset the pine pulpwood value to their starting values and increased the HSI flow targets and upper threshold by 1,000 acres (*model = “10*,*2000; 20*,*2000; 30*,*2000”*, *thlo = 1000*, *thhi = 2000*). We repeated this process four more times for 100 runs, where the maximum HSI values were set at 5,000 acres (*model = “10*,*5000; 20*,*5000; 30*,*5000”*, *thlo = 1000*, *thhi = 5000*), and pulp pinewood had flow targets of 20,000 with an upper threshold of 100,000 (*model = “10*,*20000; 20*,*20000; 30*,*20000”*, *thlo = 1000*, *thhi = 100000*; Fig 4; see S2 Table for a list of run combinations). Each model was run for 10,000 iterations.

Some additional parameters need to be assigned as R objects to designate a Habplan run. Many of these parameters are carefully explained within the Habplan User Manual [49]. However, there are a few important objects that warrant further explanation for the *HabplanR* package.

1. *Npoly*: number of forest stands (polygons).
2. *Config*: configuration of the Habplan run. The configuration is a series of numbers, where each number represents a flow component and any accompanying sub-components that are to be incorporated into the Habplan run. For our case study, this is “4,0,0,0,0,0,0,0,0,0,0,0”. The first number represents number of flows/objectives (N = 4; 3 HSI flows and pulp pinewood); this number is then followed by N (here, N = 4) pairs of numbers that state any clearcut (maintain specific cut levels over time) or block size (keep block sizes within user-defined thresholds) components (coded as either 1 = true, or 0 = false). The final three numbers of the sequence are Biol1, Biol2, and Spatial Model components (see the introduction and sections 10, 11, and 12 of the Habplan User Manual for further details). Our assigned configuration shows that there are four flow components, with no additional sub-components.
3. *Iter*: number of iterations that Habplan will sample through before stopping (i.e., number of schedules that Habplan will evaluate before outputting a final management schedule).
4. *Wd*: working directory for the R session, and thus the location where Habplan will search for input files and save output files.

The R objects created to populate the Habplan flow form are compiled using the *WriteProj* function of the *HabplanR* package. Specifically, flow parameters are combined within a single R object, and then input into one of the function arguments. The *WriteProj* function provides the option to compile up to 10 separate objectives using the flow component arguments (e.g., *f1*.*comp*, *f2*.*comp*,*… f10*.*comp*; see *HabplanR Vignette* for further details [48]). The function creates a project file, which is a.xml file format that contains the run information for the Habplan program. The run information is a combination of parameters for each flow component, and the broader Habplan information (*npoly*, *config*, *iter*, and *wd*), that the function parses from objects stored in the R session’s global environment.

#### Step 4: Selecting an appropriate management schedule

We defined a management schedule as an output from Habplan. Habplan provides a list of all forest stands and an accompanying management regime assigned to each stand (S1 Table) that will produce an outcome defined by the objective targets (*model*) and the allowable deviation from those targets (*thlo*, *thhi*). We first selected an initial four management schedules from the 100 produced by prioritizing each flow component individually by averaging the output across all 35 three-year periods, providing a mean output per time period for a single flow for each schedule. The four selected schedules produced the maximum average output (or yield) for each objective respectively. We selected these schedules to provide simple selection criteria and looked at outcome differences when prioritizing certain objectives over others. Determining which schedule is preferred requires user-specified values for “success”, which we discuss in more detail below.

To evaluate spatial composition of the four selected management schedules, we created the *HabSpace* function of the *HabplanR* package. The *HabSpace* function measures spatiotemporal characteristics of habitat and only has relevance in the context of abovementioned HSI flows (i.e., the function cannot be used to look at harvested yield across the landscape). The *HabSpace* function can be run using either a *terrestrial* or *avian mode*. These modes are in reference to movement mode by the species (or set of species) of interest. If *terrestrial* is selected (*mode = “terrestrial”*), the function will take forest stands that are connected (adjacent to one another) and link them to form larger habitat patches. Therefore, any stands that are separated by barriers (such as roads or rivers), would form separate patches. The *avian* mode (*mode = avian*) is used in conjunction with an additional argument, *dist*. Users assign a “travel distance” that may be achievable by the species of interest to traverse between potential forest patches (i.e., fly between). The *dist* (distance) determines which forest stands are accessible to any other to create larger patches. The *avian* mode could thus be used to represent terrestrial species that could easily traverse the gaps in the landscapes, such as highly vagile species moving across roads or streams. Under either mode, two files are produced: the first provides a list of all patches, area of each patch, and year that patch is calculated for (between 1-*nyear*); and the second is a summary of habitat patches for each year (year, number of patches, minimum patch size, maximum patch size, mean patch size, and total habitat area).

Separately, the *HabSpace* function applies the *calculate_lsm* function of the *landscapemetrics* R package [50] to calculate landscape metrics at a user-defined level (patch, landscape, or class). Therefore, we provide a *level* argument, that is used in the same manner as the *calculate_lsm* function of *landscapemetrics*, to assign a patch, landscape, or class-level analysis. Incorporation of the *landscapemetrics* package is included as a means to provide more comprehensive landscape metrics to the output landscape and is not linked to the *terrestrial* and *avian* modes described above.

To assess landscape configuration of each solution we used the *HabSpace* function, and aggregated patches within the landscape to highlight degree of landscape contiguity for each species. For our open-canopy and intermediate-canopy-associated species, we aggregated forest stands that were directly touching one another (stands that could be accessed without the need to cross any major landscape barrier [e.g., road]; *mode = terrestrial*). However, for our closed-canopy-associated species, we aggregated all forest stands that were within 500 m of one another into a patch and, therefore, easily flown to (*mode = avian*) by an aerial species. We chose two different modes to highlight using the *HabSpace* function, but also add to the complexity of our multiple objectives. We provide these analyses as examples. The species examples refer to broad species types and do not encompass complexities in natural history and movement ecology. We refer to the four management schedules to be compared, which we selected based on greatest mean output across the entire projected study period (35 three-year periods [105 years total]; Fig 3; Table 1), as *solutions* hereafter.

**Table 1:** Selected Habplan solutions determined via the maximum mean yield/output from Habplan flow files across the projected 35 three-year study period. Bold values depict the solution value that benefits that flow objective the most. Percentages in parentheses represent the deviation of the total flow from the maximum output possible.

Solution	Mean closed-canopy forest (acres)	Mean open-canopy forest (acres)	Mean intermediate-canopy forest (acres)	Total pine pulpwood yield (tons)
1	**10,353 (0%)**	2,160 (-63.9%)	9,912 (-0.6%)	63,476 (-86.1%)
2	3,901 (-62.3%)	**5,983 (0%)**	9,480 (-4.9%)	427,474 (-6.3%)
3	10,246 (-1%)	2,142 (-64.2%)	**9,975 (0%)**	66,136 (-85.5%)
4	3,796 (-63.3%)	5,878 (-1.7%)	9,738 (-2.4%)	**456,238 (0%)**

See the *HabplanR Vignette* [48] for additional functions developed with the R package, including options for visualizing flow outputs from Habplan management schedules that best meet objective targets, and a method for saving output schedules to.*shp* files.

## Results

### Solution one

**Fig 5:**
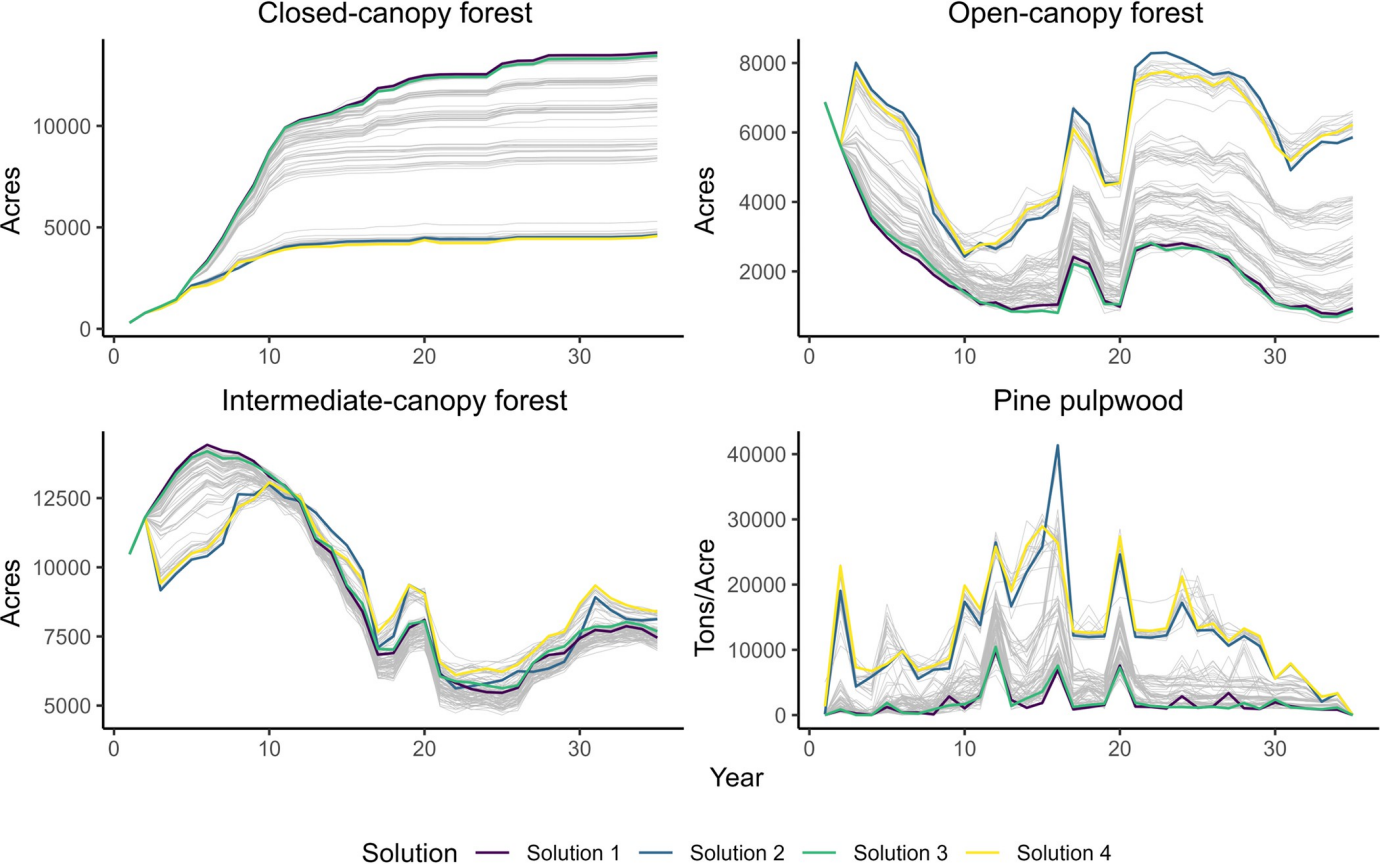
Flow output resulting from specific management schedules for each flow component across 100 runs of Habplan. Grey lines indicate the output from an individual Habplan run. The four colors represented by the figure legend depict the four selected possible solutions (i.e., selected management schedule to compare).

The first solution had *model* target for the HSI flows and *thhi* of 5,000 acres, and a *thlo* of 1,000 acres which was maintained for all model runs (*model = “10*,*5000; 20*,*5000; 30*,*5000”*, *thlo = 1000*, *thhi =* 5000), whereas targets for the pine pulpwood yield were set to 10,000 tons, with a *thhi* of 50,000 tons (*model = “10*,*10000; 20*,*10000; 30*,*10000”*, *thlo = 1000*, *thhi = 50000*). Using these parameters maximized forest acres available for closed-canopy-associated species suggesting a mean habitat output of 10,353 acres for each 3-year period. This solution resulted in the lowest yield of pine pulpwood at 63,476 tons (86.1% lower than maximum out of the four selected solutions), and the second lowest forest output for open-canopy-associated species of 2,160 acres (63.9% lower). This solution provided the second highest output for intermediate-canopy-associated species of 9,912 acres. However, the solutions did not differ greatly for total output for this species type (maximum of 4.9% deviation; Table 1; Fig 5).

### Solution two

Solution two had *model* target for HSI flows and *thhi* of 1,000 acres (*model = “10*,*1000; 20*,*1000; 30*,*1000”*, *thlo = 1000*, *thhi =* 1000), and targets for the pine pulpwood yield were set to 14,000 tons, with a *thhi* of 70,000 tons (*model = “10*,*14000; 20*,*14000; 30*,*14000”*, *thlo = 1000*, *thhi = 70000*). Using these parameters maximized open canopy conditions, with a mean forest output of 5,983 acres, while resulting in the lowest output for intermediate-canopy-associated species of 9,480 acres (4.9% lower) and the second lowest for closed-canopy-associated species of 3,901 acres (62.3% lower). However, using this management solution provided the second highest pine pulpwood yield from the four solutions selected at 427,474 tons (6.3% lower; Table 1; Fig 5).

### Solution three

Solution three had *model* target for HSI flows and *thhi* of 5,000 acres (*model = “10*,*5000; 20*,*5000; 30*,*5000”*, *thlo = 1000*, *thhi =* 5000), whereas targets for the pine pulpwood yield were set to 14,000 tons, with a *thhi* of 70,000 tons (*model = “10*,*14000; 20*,*14000; 30*,*14000”*, *thlo = 1000*, *thhi = 70000*). These parameters maximized the amount of forest available for intermediate-canopy-associated species with a mean habitat output of 9,975 acres. This solution provided a very similar overall flow output as solution one, where forest output for open-canopy associated species, and the yield for pine pulpwood were jeopardized (2,142 acres, 66,136 tons; 64.2% and 85.5% lower, respectively). The total forest output for closed-canopy-associated species using this solution was only 1% lower than solution one at 10,246 acres (Table 1; Fig 5).

### Solution four

The last solution had *model* target for the HSI flows and *thhi* of 1,000 acres (*model = “10*,*1000; 20*,*1000; 30*,*1000”*, *thlo = 1000*, *thhi =* 1000), and targets for pine pulpwood yield were set to 16,000 tons, with a *thhi* of 80,000 tons (*model = “10*,*16000; 20*,*16000; 30*,*16000”*, *thlo = 1000*, *thhi = 80000*). Using these parameters maximized yield of pine pulpwood, resulting in 456,238 tons harvested. This management solution resulted in the lowest habitat output for closed-canopy species of 3,796 acres (63.3% lower). However, output for open and intermediate-canopy-associated species were only reduced by 1.7% (5,878 acres) and 2.4% (9,738 acres), respectively (Table 1; Fig 5).

### Differences in solutions

Differences across the solutions demonstrate that selecting one path forward often requires compromises to other objectives. For example, solutions one and three created an average of >9,000 acres of habitat across the entire projection period for closed-canopy and intermediate-canopy-associated species. However, these solutions did not produce much habitat for open-canopy-associated species, only creating an average of ~2,100 acres of open-canopy conditions. The best solution for open-canopy-associated species (solution two) barely achieved just under 6,000 acres of open-canopy forest, and thus reducing this amount by greater than 60% (as happened under solution one or three) could have negative effects on open-canopy species. Additionally, using solution one or three reduced the maximum amount of pine pulpwood harvested by greater than 80%. If a manager had no need to prioritize forest for open-canopy-associated species, or revenue (harvested pine pulpwood), then solution one or three could be viable options for management as both offered opportunities to produce large expanses of forests for closed-canopy and intermediate-canopy-associated species. On the other hand, solutions two and four reduced mean closed-canopy habitat acreage by more than 60%, similar to solution one or three’s effect on open-canopy forest. However, these large reductions in overall closed-canopy forest still resulted in over an average of 3,700 acres for each solution. The outputs for solutions two and four were very similar, although solution two slightly prioritized area for open and closed-canopy-associated species over intermediate-canopy-associated species and revenue. This was reversed for solution four.

### Spatial comparisons

Using direct comparisons of total output for each flow, and the perspective of a forest manager interested in revenue, we determined that the resulting overall loss of yield for pine pulpwood flow was too great for solutions one and three (86% and 85.5%, respectively). We therefore performed spatial analysis on solutions two and four only to show that, even for similar solutions in terms of overall output, spatial differences can occur (Table 2 and Fig 5).

**Table 2:** Patch-level landscape metrics derived using the *HabSpace* function of *HabplanR* for the entire 35 three-year projection period, across two possible Habplan solutions. Reported metrics include total forest area within the specific canopy openness category (acres, mean number of patches, and mean patch area [acres]). Values within parentheses represent the range of mean values across the 35 three-year projection period.

Solution	Species	Total area (acres)	Number of patches	Patch area (acres)
2	Closed	3901 (301–4822)	102 (12–120)	37.1 (25.2–40.3)
2	Open	5983 (2447–8806)	75 (45–104)	97.1 (44.2–115.9)
2	Intermediate	9480 (5742–13619)	107 (78–144)	92.2 (50.4–171)
4	Closed	3796 (301–4760)	83 (12–97)	43.7 (25.2–49.2)
4	Open	5878 (2539–8266)	85 (43–110)	68.7 (41.3–87.5)
4	Intermediate	9738 (6218–13693)	103 (79–148)	97.1 (52.9–162.8)

We assessed three patch-level (grouped forest stands containing desired habitat qualities based on terrestrial or avian movement modes) landscape metrics across the three species types for our two selected solutions: total area, number of patches, and mean patch area. Both solutions produced very similar patch metric values in our example. However, there were some differences that aided in a final solution decision. First, solution two provided more area for closed-canopy-associated species on average and reached greater total area throughout the entire projection period (Table 2). Additionally, solution two created fewer habitat patches on average for open-canopy-associated species than solution four, where patches also consisted of a greater area. Alternatively, solution four projected more forest area for intermediate-canopy-associated species. Solution four also produced fewer habitat patches for closed and intermediate-canopy-associated species (Table 2), where the mean area of patches was also greater. Although the mean total habitat area for open-canopy species across the projection period was lower when using solution four, this area was always maintained at greater than 2,500 acres for any individual three-year period, which was not the case for solution two (2446.6 acres; Table 2).

**Fig 6:**
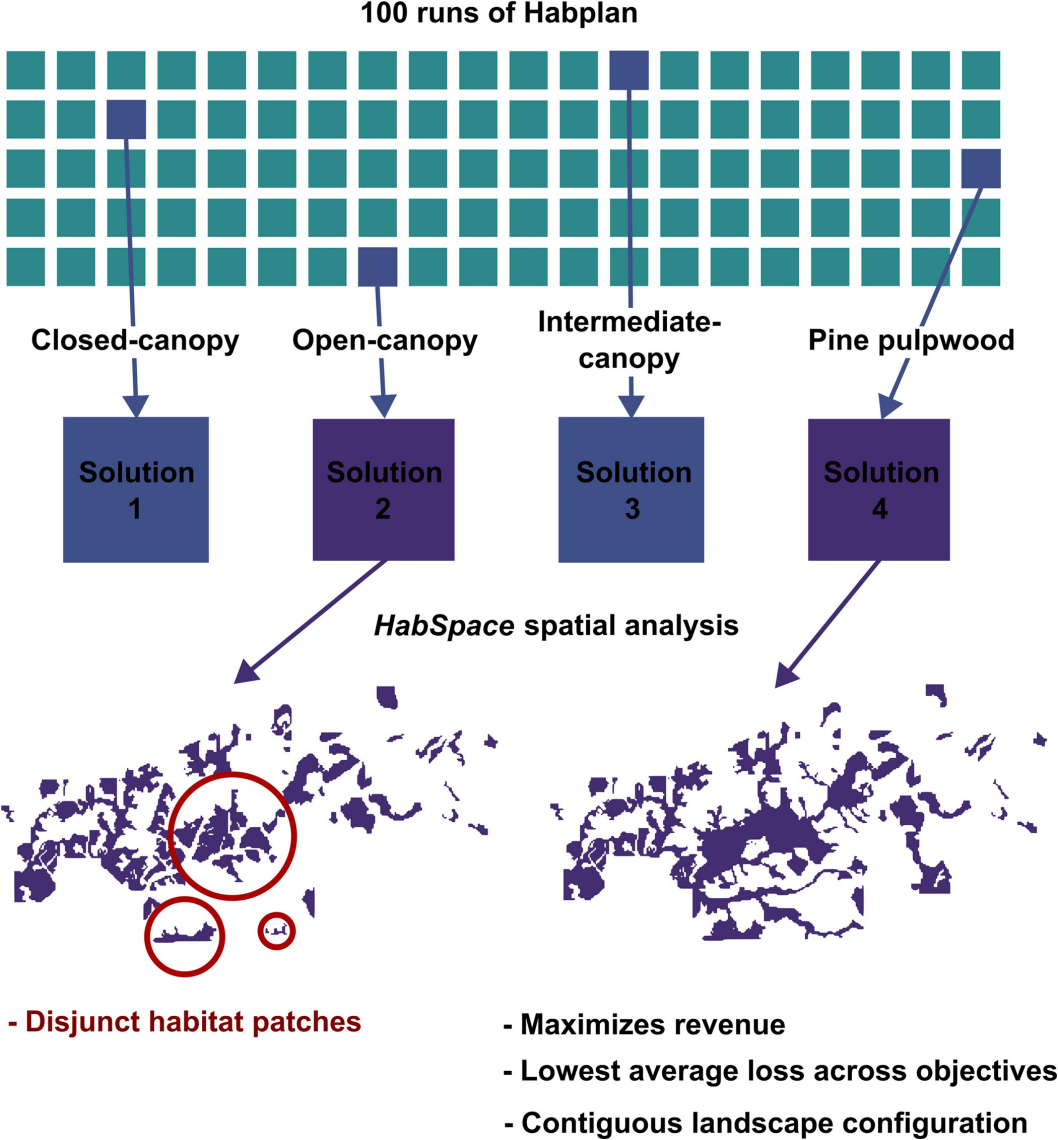
Example framework for selecting an appropriate management solution for multi-species/objective forestry management.

Solution four produced large expanses of contiguous habitat for open-canopy-associated species, evidenced in time periods 5, 25, and 35 of the projections (Fig 5). Although solution two created a larger total habitat area on average for open-canopy-associated species, and fewer patches (Table 2), the positioning of those patches created highly disjunct habitat areas (Fig 5). The disjunct nature of patches created for closed-canopy-associated species by solution four were more appropriate for bird species that can more readily move between them. For intermediate-canopy-associated species, both solutions created similar landscape metric values, with large habitat patches on average (Table 2), which can be seen across large habitat areas created (Fig 5). For this latter species, harvest scheduling program users may want to consider timing of how different habitat patches are connected and longevity of larger patches. However, for our example, the differences were negligible, and the habitat area was large regardless, and more weight was given to the open and closed-canopy habitat arrangements. Based on the culmination of these data, we chose solution four as our “optimum” management schedule (Fig 6).

## Discussion

We found that forest management schedules that promote multi-species management can be selected, but solutions must be objective-driven, and therefore, compromises to specific objectives may need to be made. Specifically, due to contradictory vegetation and forest structural needs of some wildlife species (e.g., open vs closed-canopy specialists [51]), it may not be possible to produce the highest habitat yield possible concurrently for multiple species with different structural needs. Therefore, landscapes can be prioritized to produce habitat area for multiple species guilds, but at less area than could be achieved through single species management [52].

In our case study, we used simple selection criteria as an example for comparing some highly varying solutions based on maximizing output for each objective individually. Reducing the number of options to four via this method permitted easy example comparisons of possible solutions. We demonstrated careful consideration of costs and benefits of solutions focused on different objectives, which may be needed to achieve multiple objectives. Congruent with previous studies [53], prioritizing conservation efforts for one species–in this case, maximizing forest area for closed-canopy-associated species–was contradictory to multi-species/objective management, exhibited as the substantial reduction in forest area for open-canopy-associated species and pine pulpwood yield. For our example, we attempted to balance outputs from all of our input flows. We ultimately selected the solution that maximized harvested pine pulpwood yield because this presented the most appropriate balance of habitat for our three species, while providing more appropriate spatial characteristics. This suggests that management options that give greater weight to economic objectives can still positively affect multi-species management [52]. However, a manager with different priorities may have selected a different preferred solution, demonstrating utility of examining multiple potential solutions.

**Fig 7:**
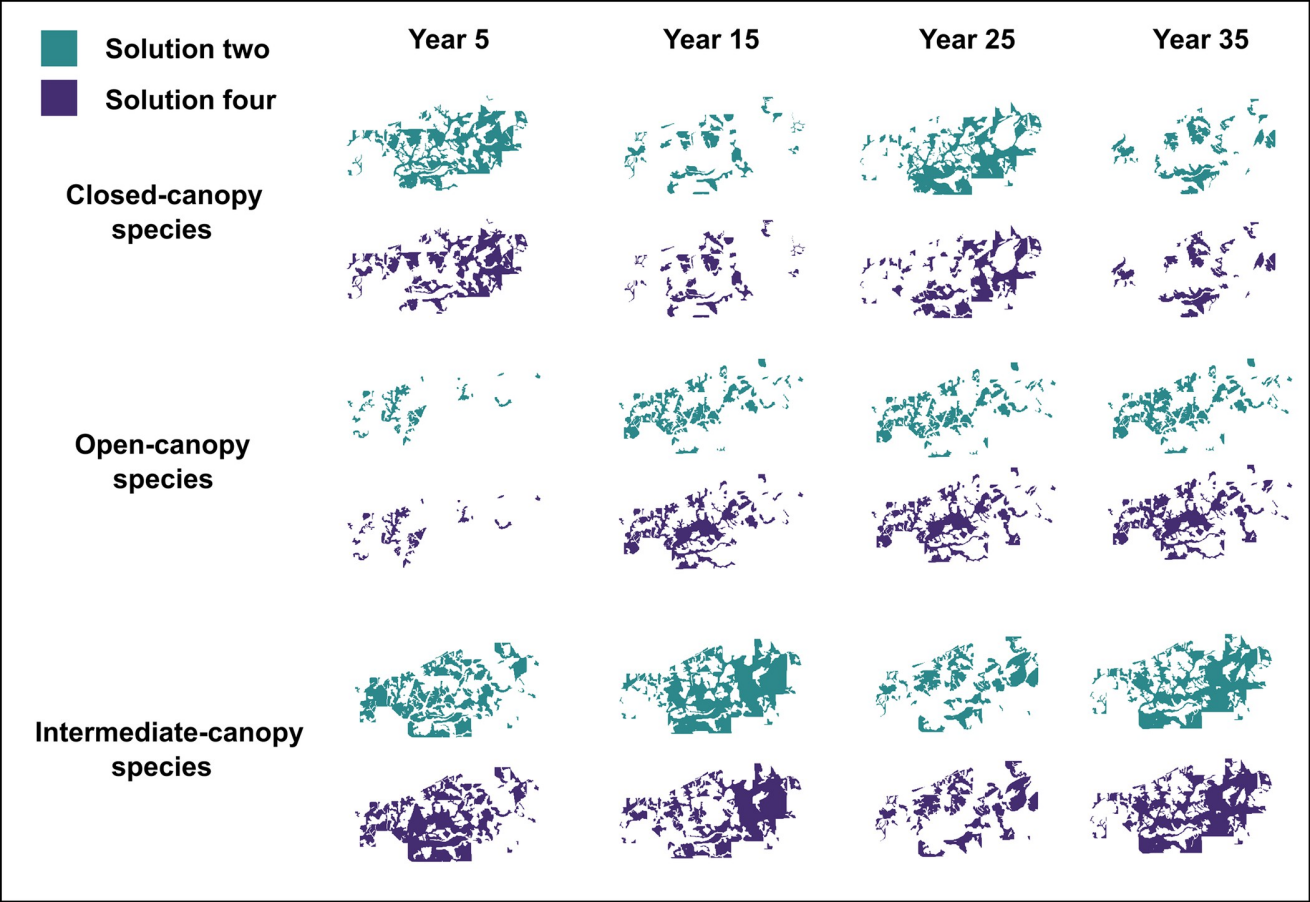
Available habitat across a portion of the study landscape projected for each species type based on selected solutions. Landscapes are projected for four time periods, T = 5, 15, 25, and 35 3-year periods. The upper, middle, and lower panels depict closed, open, and intermediate-canopy forests, respectively.

Using landscape maps is highly subjective to each case study, and their interpretation can depend on a multitude of factors. For example, a solution may be deemed unsuitable if habitat patches fall within an area bounded by roads, in the case where the intended use is to provide forest for a species that is sensitive to road mortality [54]. Alternatively, population assessments may have found low population density within a specific region of a study site, and prioritizing solutions that create large expanses of forest area across a longer period may be necessary. Furthermore, a solution may create corridors that connect larger expanses of forest to one another, permitting gene flow between previously disjunct subpopulations, and ultimately reducing effects of habitat fragmentation [55]. We performed spatial analysis via the *HabSpace* function on two solutions selected for high objective performance. In this example, we assessed landscape configuration of each solution based solely on habitat patches they created. Out of these final solutions, solution two resulted in a larger habitat area for open-canopy-associated species and fewer patches, but the configuration of patches resulted in a spatially disjunct landscape (Fig 7). Landscape configuration is an important consideration for wildlife conservation and other ecological objectives [56, 57], and can be a more important factor for some species than the amount of habitat area [58, 59].

The needs of some species and taxa are complex and managers require in-depth knowledge about their study systems and conservation goals to select the most appropriate management schedule. Forest managers may have much more specific criteria for narrowing the search for the preferred management schedule. For example, Martin et al. [7] developed models that needed to attain specific timber harvest volume targets but narrowed their framework to improve habitat area and connectivity for woodland caribou (*Rangifer tarandus caribou*). Additionally, [60] developed a heuristic spatial forest planning process that tackled a complex multi-objective scenario to maintain forest structure, maximize revenue, and maintain a minimum habitat area for threatened northern spotted owls (*Strix occidentalis caurina*) in Oregon, United States. We decided that solutions one and three resulted in too large a loss of open canopy forest acres and harvested pine pulpwood to deem as viable management options. However, these may be appropriate for forest planners who are managing specific species over others [60].

There are many ways solutions can be filtered to select a solution for a forest manager’s specific needs, including through discussion with partners, other managers, and possibly via public forum to decide important factors influencing future management priorities [12]. We provide a basic pathway for using forest management and spatial landscape tools to determine a potentially viable solution for achieving multiple objectives. Habplan iteratively finds new scheduling solutions until the maximum number of iterations is reached, or the program is manually stopped [28]. These solutions do not signify the “best” possible solution (i.e., maximizing all objectives) but instead compute schedules that can achieve desired targets (within a user-defined upper and lower threshold). Therefore, this forest management software can provide multiple solutions to the same management problem to achieve quantitative targets, all of which may present either a slightly or vastly different set of regimes applied to each stand, which may change the resulting spatial configuration of the landscape. This provides multiple management options at both a quantitative and spatial level, both of which are valuable tools to meet the complex (and often contrasting) needs of multiple objectives.

Considering the flexibility of HSI values (such as generalist vs specialist species) could help forest managers decide on their preferred management schedule. Additionally, we propose that the landscape configuration effects of different management schedules can be compared to provide a more holistic interpretation of different objective outcomes and can support the decision-making process for forest management practices [61, 62].

## Supporting information

S1 Fig Relationship between individual flow outputs for each possible management solution (Solution 1–4). Each colored point represents the flow values for a unique solution.
(TIFF)

S1 Table Descriptions of management regimes and their corresponding regime ID, used within the Forest Vegetation Simulator growth model to project stand conditions. BA: Basal Area, LL: longleaf pine (*Pinus palustris*), TPA: Trees Per Acre.
(DOCX)

S2 Table All Habplan model runs. All HSI flows were treated the same. Year 10, 20, and 30 show the targets for their respective three-year time period (10, 20, 30). The upper and lower threshold are represented as Thhi and Thlo, respectively. These thresholds represent the value that Habplan can deviate away from the targets. The Run values depicted with an * show the “Solutions” discussed in the results (solution 1: 90; solution 2: 16; solution 3: 94; solution 4: 16).
(DOCX)
