# Supplementary figures and images for: A multi-objective approach for timber harvest scheduling to include management of at-risk species and spatial configuration objectives
Source: PLoS One. 2024 Oct 25;19(10):e0302640. doi: 10.1371/journal.pone.0302640 (PMC11508488; doi:10.1371/journal.pone.0302640)

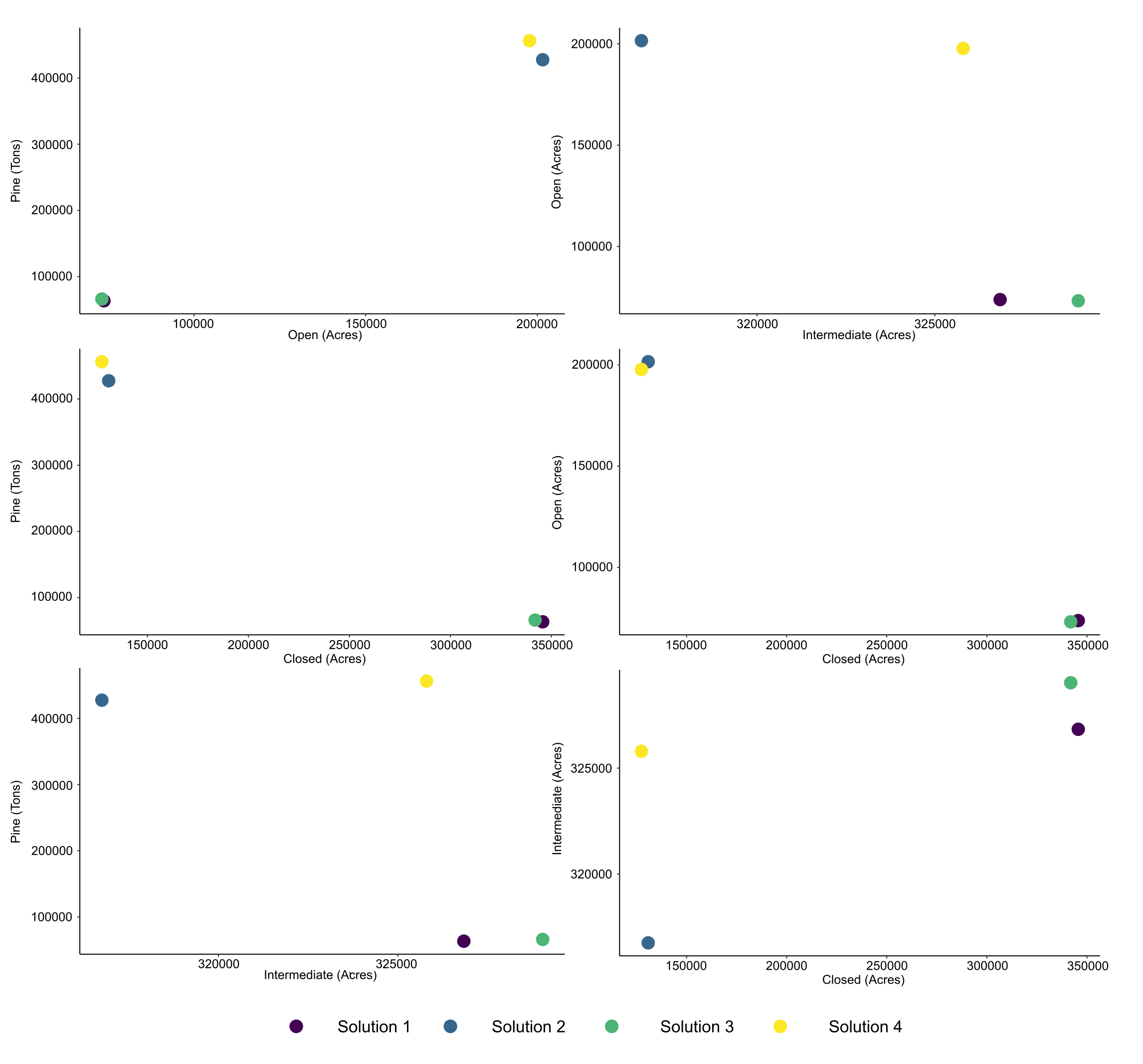

Supplement: S1 Fig — Each colored point represents the flow values for a unique solution. (TIFF) [file pone.0302640.s001.tiff]
