# Supplementary material for: A multi-objective approach for timber harvest scheduling to include management of at-risk species and spatial configuration objectives
Source: PLoS One. 2024 Oct 25;19(10):e0302640. doi: 10.1371/journal.pone.0302640 (PMC11508488; doi:10.1371/journal.pone.0302640)
Supplement: S1 Table — BA: Basal Area, LL: longleaf pine (Pinus palustris), TPA: Trees Per Acre. (DOCX) [file pone.0302640.s002.docx]

| Regime ID | | Label | Description |
| --- | --- | --- | --- |
| 0 | No harvest | | No harvesting activity |
| 1 | Interplant LL at start | | Thin to 20 BA in period 1. Plant 600 LL TPA. First thin @ 100 BA to 70 BA, second thin 12 years later to 50 BA, third thin 12 years later to 30 BA. |
| 2 | Interplant LL #1 | | Thin @ 150 BA to 20 BA. Plant 600 LL TPA. Thin 3X as in Regime 1. |
| 3 | Interplant LL #2 | | Thin @ 120 BA to 20 BA. Plant 600 LL TPA. Thin 3X as in Regime 1. |
| 4 | Three thins #1 | | First thin @ 120 BA to 70 BA. Second thin 12 years later to 50 BA. Third thin @ 95 BA ft to 30 BA. |
| 5 | Three thins #2 | | First thin @ 140 BA to 70 BA. Second thin 12 years later to 50 BA. Third thin @ 95 BA ft to 30 BA. |
| 6 | Three thins and interplant #1 | | First thin @ 90 BA to 60 BA, second thin 12 years later to 50 BA, third thin 12 years later to 20 BA. Plant 600 LL TPA. First LL thin @ 100 BA to 70 BA, second LL thin @ 90 BA to 50 BA. Third LL thin @ 60 BA to 30 BA. |
| 7 | Three thins and interplant #2 | | First thin @ 100 BA to 70 BA, followed by second and third thinnings as in regime 6. Plant 600 LL TPA. First LL thin @ 110 BA to 70 BA, second LL thin @ 105 BA to 50 BA, third LL thin @ 80 BA to 30 BA. |
| 8 | Clearcut and plant LL #1 | | Clearcut in period 1. Plant 600 LL TPA. First thin @ 100 BA to 70 BA, second thin @ 90 BA to 50 BA, third thin @ 60 BA to 30 BA. |
| 9 | Clearcut and plant LL #2 | | Clearcut in period 4. Plant 600 LL TPA. Thin 3X as in regime 8. |
