# Supplementary material for: A multi-objective approach for timber harvest scheduling to include management of at-risk species and spatial configuration objectives
Source: PLoS One. 2024 Oct 25;19(10):e0302640. doi: 10.1371/journal.pone.0302640 (PMC11508488; doi:10.1371/journal.pone.0302640)
Supplement: S2 Table — All HSI flows were treated the same. Year 10, 20, and 30 show the targets for their respective three-year time period (10, 20, 30). The upper and lower threshold are represented as Thhi and Thlo, respectively. These thresholds represent the value that Habplan can deviate away from the targets. The Run values depicted with an * show the “Solutions” discussed in the results (solution 1: 90; solution 2: 16; solution 3: 94; solution 4: 16). (DOCX) [file pone.0302640.s003.docx]

|  | **HSI flows** | | | | | **Pine Pulpwood flow** | | | | |
| --- | --- | --- | --- | --- | --- | --- | --- | --- | --- | --- |
| **Run** | **Thlo** | **Thhi** | **Year 10** | **Year 20** | **Year 30** | **Thlo** | **Thhi** | **Year 10** | **Year 20** | **Year 30** |
| 1 | 1000 | 1000 | 1000 | 1000 | 1000 | 1000 | 5000 | 1000 | 1000 | 1000 |
| 2 | 1000 | 1000 | 1000 | 1000 | 1000 | 1000 | 10000 | 2000 | 2000 | 2000 |
| 3 | 1000 | 1000 | 1000 | 1000 | 1000 | 1000 | 15000 | 3000 | 3000 | 3000 |
| 4 | 1000 | 1000 | 1000 | 1000 | 1000 | 1000 | 20000 | 4000 | 4000 | 4000 |
| 5 | 1000 | 1000 | 1000 | 1000 | 1000 | 1000 | 25000 | 5000 | 5000 | 5000 |
| 6 | 1000 | 1000 | 1000 | 1000 | 1000 | 1000 | 30000 | 6000 | 6000 | 6000 |
| 7 | 1000 | 1000 | 1000 | 1000 | 1000 | 1000 | 35000 | 7000 | 7000 | 7000 |
| 8 | 1000 | 1000 | 1000 | 1000 | 1000 | 1000 | 40000 | 8000 | 8000 | 8000 |
| 9 | 1000 | 1000 | 1000 | 1000 | 1000 | 1000 | 45000 | 9000 | 9000 | 9000 |
| 10 | 1000 | 1000 | 1000 | 1000 | 1000 | 1000 | 50000 | 10000 | 10000 | 10000 |
| 11 | 1000 | 1000 | 1000 | 1000 | 1000 | 1000 | 55000 | 11000 | 11000 | 11000 |
| 12 | 1000 | 1000 | 1000 | 1000 | 1000 | 1000 | 60000 | 12000 | 12000 | 12000 |
| 13 | 1000 | 1000 | 1000 | 1000 | 1000 | 1000 | 65000 | 13000 | 13000 | 13000 |
| 14^*^ | 1000 | 1000 | 1000 | 1000 | 1000 | 1000 | 70000 | 14000 | 14000 | 14000 |
| 15 | 1000 | 1000 | 1000 | 1000 | 1000 | 1000 | 75000 | 15000 | 15000 | 15000 |
| 16^*^ | 1000 | 1000 | 1000 | 1000 | 1000 | 1000 | 80000 | 16000 | 16000 | 16000 |
| 17 | 1000 | 1000 | 1000 | 1000 | 1000 | 1000 | 85000 | 17000 | 17000 | 17000 |
| 18 | 1000 | 1000 | 1000 | 1000 | 1000 | 1000 | 90000 | 18000 | 18000 | 18000 |
| 19 | 1000 | 1000 | 1000 | 1000 | 1000 | 1000 | 95000 | 19000 | 19000 | 19000 |
| 20 | 1000 | 1000 | 1000 | 1000 | 1000 | 1000 | 100000 | 20000 | 20000 | 20000 |
| 21 | 1000 | 2000 | 2000 | 2000 | 2000 | 1000 | 5000 | 1000 | 1000 | 1000 |
| 22 | 1000 | 2000 | 2000 | 2000 | 2000 | 1000 | 10000 | 2000 | 2000 | 2000 |
| 23 | 1000 | 2000 | 2000 | 2000 | 2000 | 1000 | 15000 | 3000 | 3000 | 3000 |
| 24 | 1000 | 2000 | 2000 | 2000 | 2000 | 1000 | 20000 | 4000 | 4000 | 4000 |
| 25 | 1000 | 2000 | 2000 | 2000 | 2000 | 1000 | 25000 | 5000 | 5000 | 5000 |
| 26 | 1000 | 2000 | 2000 | 2000 | 2000 | 1000 | 30000 | 6000 | 6000 | 6000 |
| 27 | 1000 | 2000 | 2000 | 2000 | 2000 | 1000 | 35000 | 7000 | 7000 | 7000 |
| 28 | 1000 | 2000 | 2000 | 2000 | 2000 | 1000 | 40000 | 8000 | 8000 | 8000 |
| 29 | 1000 | 2000 | 2000 | 2000 | 2000 | 1000 | 45000 | 9000 | 9000 | 9000 |
| 30 | 1000 | 2000 | 2000 | 2000 | 2000 | 1000 | 50000 | 10000 | 10000 | 10000 |
| 31 | 1000 | 2000 | 2000 | 2000 | 2000 | 1000 | 55000 | 11000 | 11000 | 11000 |
| 32 | 1000 | 2000 | 2000 | 2000 | 2000 | 1000 | 60000 | 12000 | 12000 | 12000 |
| 33 | 1000 | 2000 | 2000 | 2000 | 2000 | 1000 | 65000 | 13000 | 13000 | 13000 |
| 34 | 1000 | 2000 | 2000 | 2000 | 2000 | 1000 | 70000 | 14000 | 14000 | 14000 |
| 35 | 1000 | 2000 | 2000 | 2000 | 2000 | 1000 | 75000 | 15000 | 15000 | 15000 |
| 36 | 1000 | 2000 | 2000 | 2000 | 2000 | 1000 | 80000 | 16000 | 16000 | 16000 |
| 37 | 1000 | 2000 | 2000 | 2000 | 2000 | 1000 | 85000 | 17000 | 17000 | 17000 |
| 38 | 1000 | 2000 | 2000 | 2000 | 2000 | 1000 | 90000 | 18000 | 18000 | 18000 |
| 39 | 1000 | 2000 | 2000 | 2000 | 2000 | 1000 | 95000 | 19000 | 19000 | 19000 |
| 40 | 1000 | 2000 | 2000 | 2000 | 2000 | 1000 | 100000 | 20000 | 20000 | 20000 |
| 41 | 1000 | 3000 | 3000 | 3000 | 3000 | 1000 | 5000 | 1000 | 1000 | 1000 |
| 42 | 1000 | 3000 | 3000 | 3000 | 3000 | 1000 | 10000 | 2000 | 2000 | 2000 |
| 43 | 1000 | 3000 | 3000 | 3000 | 3000 | 1000 | 15000 | 3000 | 3000 | 3000 |
| 44 | 1000 | 3000 | 3000 | 3000 | 3000 | 1000 | 20000 | 4000 | 4000 | 4000 |
| 45 | 1000 | 3000 | 3000 | 3000 | 3000 | 1000 | 25000 | 5000 | 5000 | 5000 |
| 46 | 1000 | 3000 | 3000 | 3000 | 3000 | 1000 | 30000 | 6000 | 6000 | 6000 |
| 47 | 1000 | 3000 | 3000 | 3000 | 3000 | 1000 | 35000 | 7000 | 7000 | 7000 |
| 48 | 1000 | 3000 | 3000 | 3000 | 3000 | 1000 | 40000 | 8000 | 8000 | 8000 |
| 49 | 1000 | 3000 | 3000 | 3000 | 3000 | 1000 | 45000 | 9000 | 9000 | 9000 |
| 50 | 1000 | 3000 | 3000 | 3000 | 3000 | 1000 | 50000 | 10000 | 10000 | 10000 |
| 51 | 1000 | 3000 | 3000 | 3000 | 3000 | 1000 | 55000 | 11000 | 11000 | 11000 |
| 52 | 1000 | 3000 | 3000 | 3000 | 3000 | 1000 | 60000 | 12000 | 12000 | 12000 |
| 53 | 1000 | 3000 | 3000 | 3000 | 3000 | 1000 | 65000 | 13000 | 13000 | 13000 |
| 54 | 1000 | 3000 | 3000 | 3000 | 3000 | 1000 | 70000 | 14000 | 14000 | 14000 |
| 55 | 1000 | 3000 | 3000 | 3000 | 3000 | 1000 | 75000 | 15000 | 15000 | 15000 |
| 56 | 1000 | 3000 | 3000 | 3000 | 3000 | 1000 | 80000 | 16000 | 16000 | 16000 |
| 57 | 1000 | 3000 | 3000 | 3000 | 3000 | 1000 | 85000 | 17000 | 17000 | 17000 |
| 58 | 1000 | 3000 | 3000 | 3000 | 3000 | 1000 | 90000 | 18000 | 18000 | 18000 |
| 59 | 1000 | 3000 | 3000 | 3000 | 3000 | 1000 | 95000 | 19000 | 19000 | 19000 |
| 60 | 1000 | 3000 | 3000 | 3000 | 3000 | 1000 | 100000 | 20000 | 20000 | 20000 |
| 61 | 1000 | 4000 | 4000 | 4000 | 4000 | 1000 | 5000 | 1000 | 1000 | 1000 |
| 62 | 1000 | 4000 | 4000 | 4000 | 4000 | 1000 | 10000 | 2000 | 2000 | 2000 |
| 63 | 1000 | 4000 | 4000 | 4000 | 4000 | 1000 | 15000 | 3000 | 3000 | 3000 |
| 64 | 1000 | 4000 | 4000 | 4000 | 4000 | 1000 | 20000 | 4000 | 4000 | 4000 |
| 65 | 1000 | 4000 | 4000 | 4000 | 4000 | 1000 | 25000 | 5000 | 5000 | 5000 |
| 66 | 1000 | 4000 | 4000 | 4000 | 4000 | 1000 | 30000 | 6000 | 6000 | 6000 |
| 67 | 1000 | 4000 | 4000 | 4000 | 4000 | 1000 | 35000 | 7000 | 7000 | 7000 |
| 68 | 1000 | 4000 | 4000 | 4000 | 4000 | 1000 | 40000 | 8000 | 8000 | 8000 |
| 69 | 1000 | 4000 | 4000 | 4000 | 4000 | 1000 | 45000 | 9000 | 9000 | 9000 |
| 70 | 1000 | 4000 | 4000 | 4000 | 4000 | 1000 | 50000 | 10000 | 10000 | 10000 |
| 71 | 1000 | 4000 | 4000 | 4000 | 4000 | 1000 | 55000 | 11000 | 11000 | 11000 |
| 72 | 1000 | 4000 | 4000 | 4000 | 4000 | 1000 | 60000 | 12000 | 12000 | 12000 |
| 73 | 1000 | 4000 | 4000 | 4000 | 4000 | 1000 | 65000 | 13000 | 13000 | 13000 |
| 74 | 1000 | 4000 | 4000 | 4000 | 4000 | 1000 | 70000 | 14000 | 14000 | 14000 |
| 75 | 1000 | 4000 | 4000 | 4000 | 4000 | 1000 | 75000 | 15000 | 15000 | 15000 |
| 76 | 1000 | 4000 | 4000 | 4000 | 4000 | 1000 | 80000 | 16000 | 16000 | 16000 |
| 77 | 1000 | 4000 | 4000 | 4000 | 4000 | 1000 | 85000 | 17000 | 17000 | 17000 |
| 78 | 1000 | 4000 | 4000 | 4000 | 4000 | 1000 | 90000 | 18000 | 18000 | 18000 |
| 79 | 1000 | 4000 | 4000 | 4000 | 4000 | 1000 | 95000 | 19000 | 19000 | 19000 |
| 80 | 1000 | 4000 | 4000 | 4000 | 4000 | 1000 | 100000 | 20000 | 20000 | 20000 |
| 81 | 1000 | 5000 | 5000 | 5000 | 5000 | 1000 | 5000 | 1000 | 1000 | 1000 |
| 82 | 1000 | 5000 | 5000 | 5000 | 5000 | 1000 | 10000 | 2000 | 2000 | 2000 |
| 83 | 1000 | 5000 | 5000 | 5000 | 5000 | 1000 | 15000 | 3000 | 3000 | 3000 |
| 84 | 1000 | 5000 | 5000 | 5000 | 5000 | 1000 | 20000 | 4000 | 4000 | 4000 |
| 85 | 1000 | 5000 | 5000 | 5000 | 5000 | 1000 | 25000 | 5000 | 5000 | 5000 |
| 86 | 1000 | 5000 | 5000 | 5000 | 5000 | 1000 | 30000 | 6000 | 6000 | 6000 |
| 87 | 1000 | 5000 | 5000 | 5000 | 5000 | 1000 | 35000 | 7000 | 7000 | 7000 |
| 88 | 1000 | 5000 | 5000 | 5000 | 5000 | 1000 | 40000 | 8000 | 8000 | 8000 |
| 89 | 1000 | 5000 | 5000 | 5000 | 5000 | 1000 | 45000 | 9000 | 9000 | 9000 |
| 90^*^ | 1000 | 5000 | 5000 | 5000 | 5000 | 1000 | 50000 | 10000 | 10000 | 10000 |
| 91 | 1000 | 5000 | 5000 | 5000 | 5000 | 1000 | 55000 | 11000 | 11000 | 11000 |
| 92 | 1000 | 5000 | 5000 | 5000 | 5000 | 1000 | 60000 | 12000 | 12000 | 12000 |
| 93 | 1000 | 5000 | 5000 | 5000 | 5000 | 1000 | 65000 | 13000 | 13000 | 13000 |
| 94^*^ | 1000 | 5000 | 5000 | 5000 | 5000 | 1000 | 70000 | 14000 | 14000 | 14000 |
| 95 | 1000 | 5000 | 5000 | 5000 | 5000 | 1000 | 75000 | 15000 | 15000 | 15000 |
| 96 | 1000 | 5000 | 5000 | 5000 | 5000 | 1000 | 80000 | 16000 | 16000 | 16000 |
| 97 | 1000 | 5000 | 5000 | 5000 | 5000 | 1000 | 85000 | 17000 | 17000 | 17000 |
| 98 | 1000 | 5000 | 5000 | 5000 | 5000 | 1000 | 90000 | 18000 | 18000 | 18000 |
| 99 | 1000 | 5000 | 5000 | 5000 | 5000 | 1000 | 95000 | 19000 | 19000 | 19000 |
| 100 | 1000 | 5000 | 5000 | 5000 | 5000 | 1000 | 100000 | 20000 | 20000 | 20000 |
